# Supplementary material for: Utility of long-read sequencing for All of Us
Source: Nat Commun. 2024 Jan 29;15:837. doi: 10.1038/s41467-024-44804-3 (PMC10822842; doi:10.1038/s41467-024-44804-3)
Supplement: Supplementary file 1 — Supplementary information [file 41467_2024_44804_MOESM1_ESM.docx]

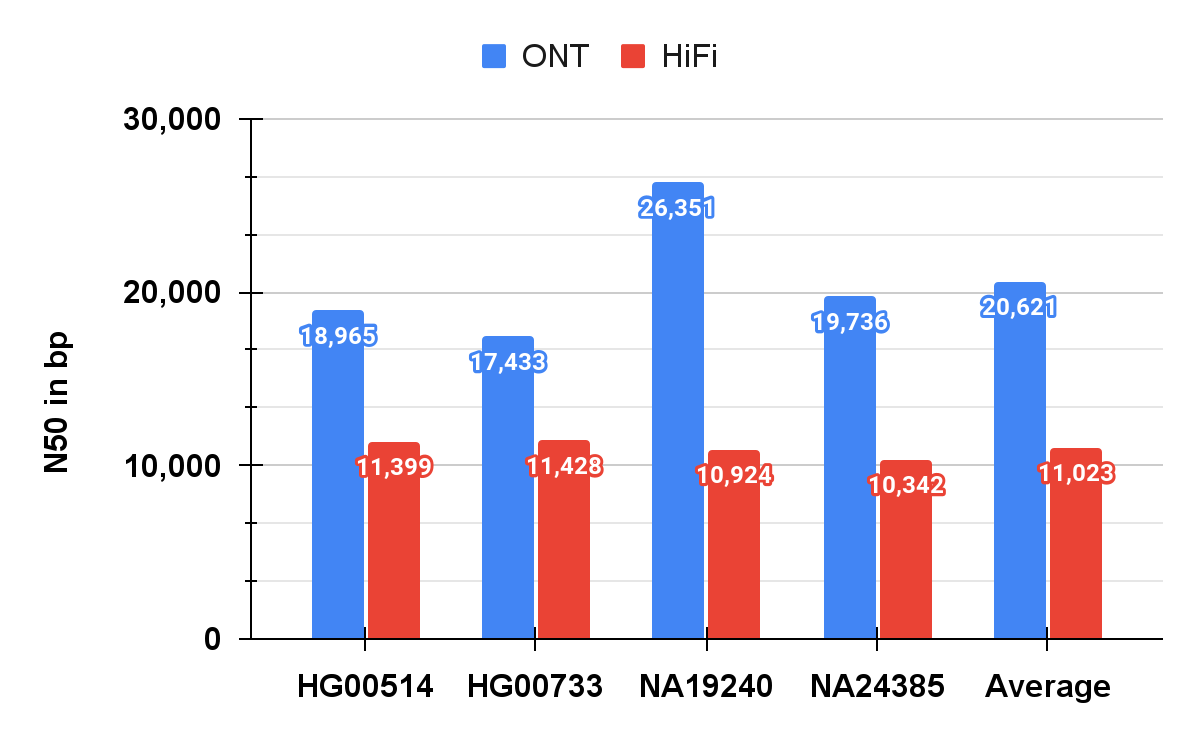


Figure 1: Mapping N50 between HapMap samples.


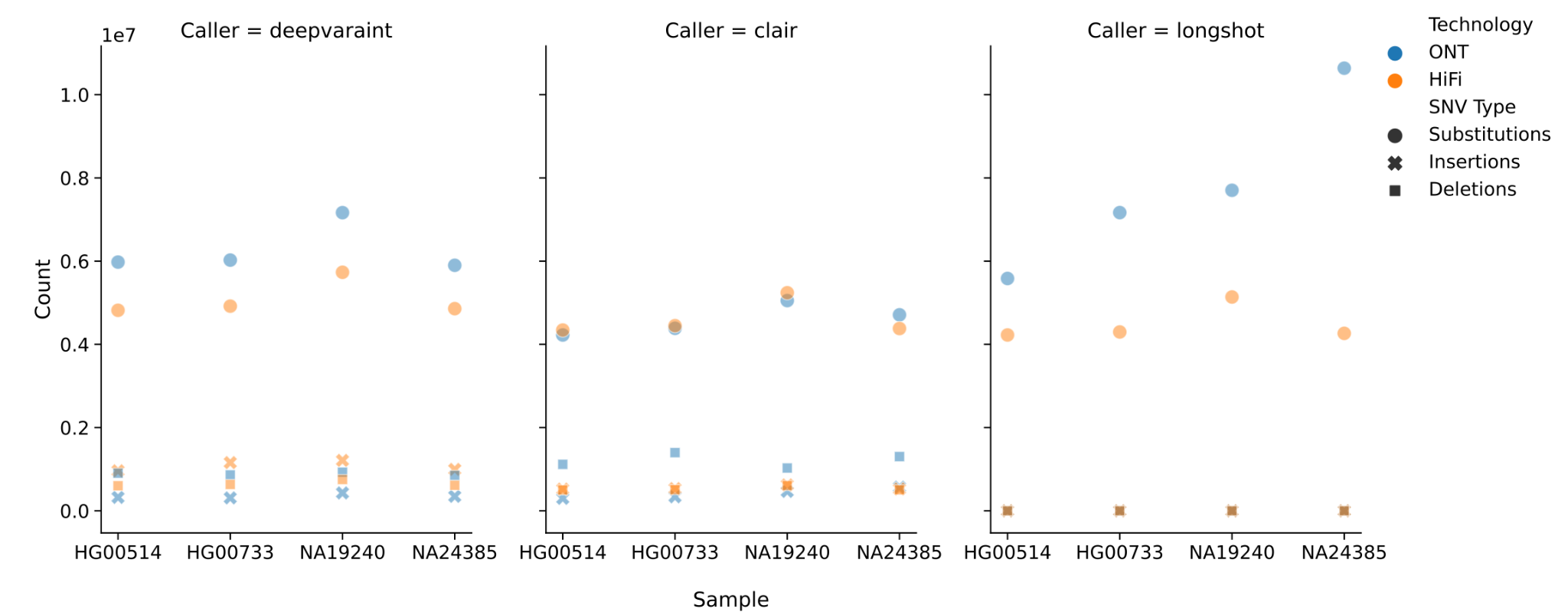


**Figure 2:** number of substitutions, insertions, and deletions identified between HiFi and ONT using both clair3 and DeepVariant.


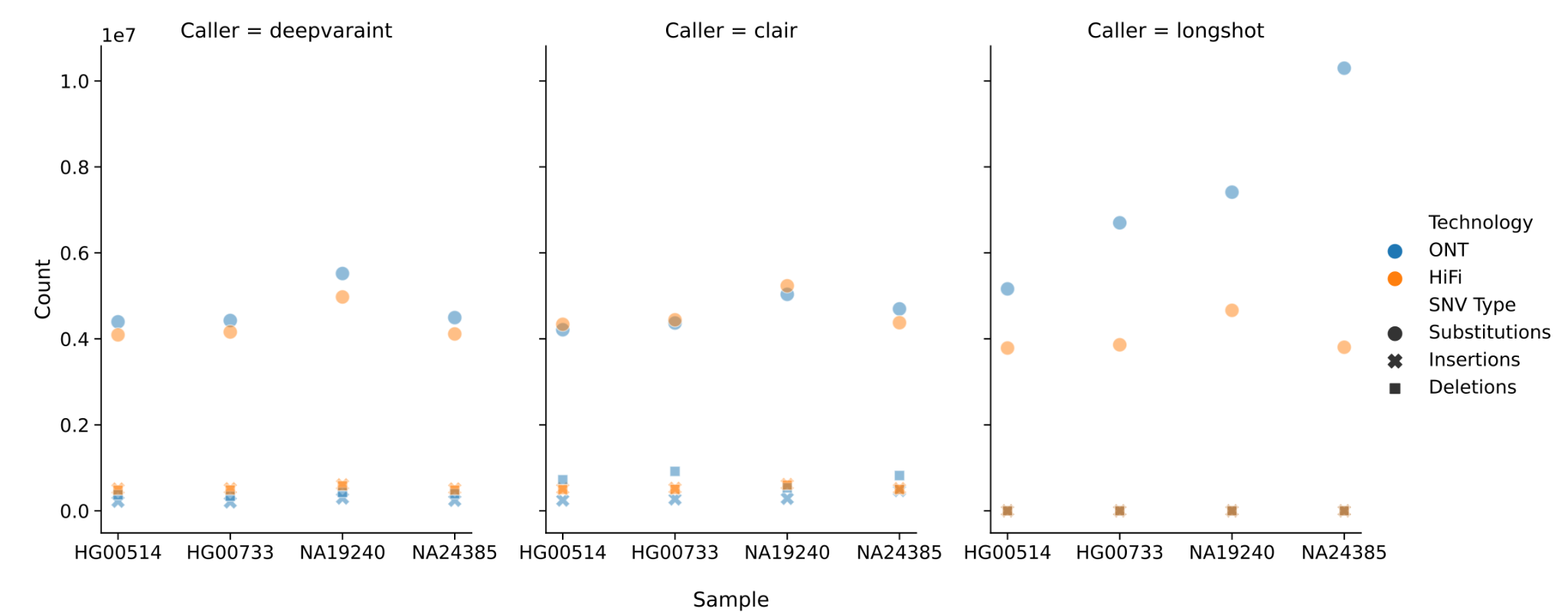


**Figure 3:** number of substitutions, insertions, and deletions identified between HiFi and ONT using both clairr3 and DeepVariant.


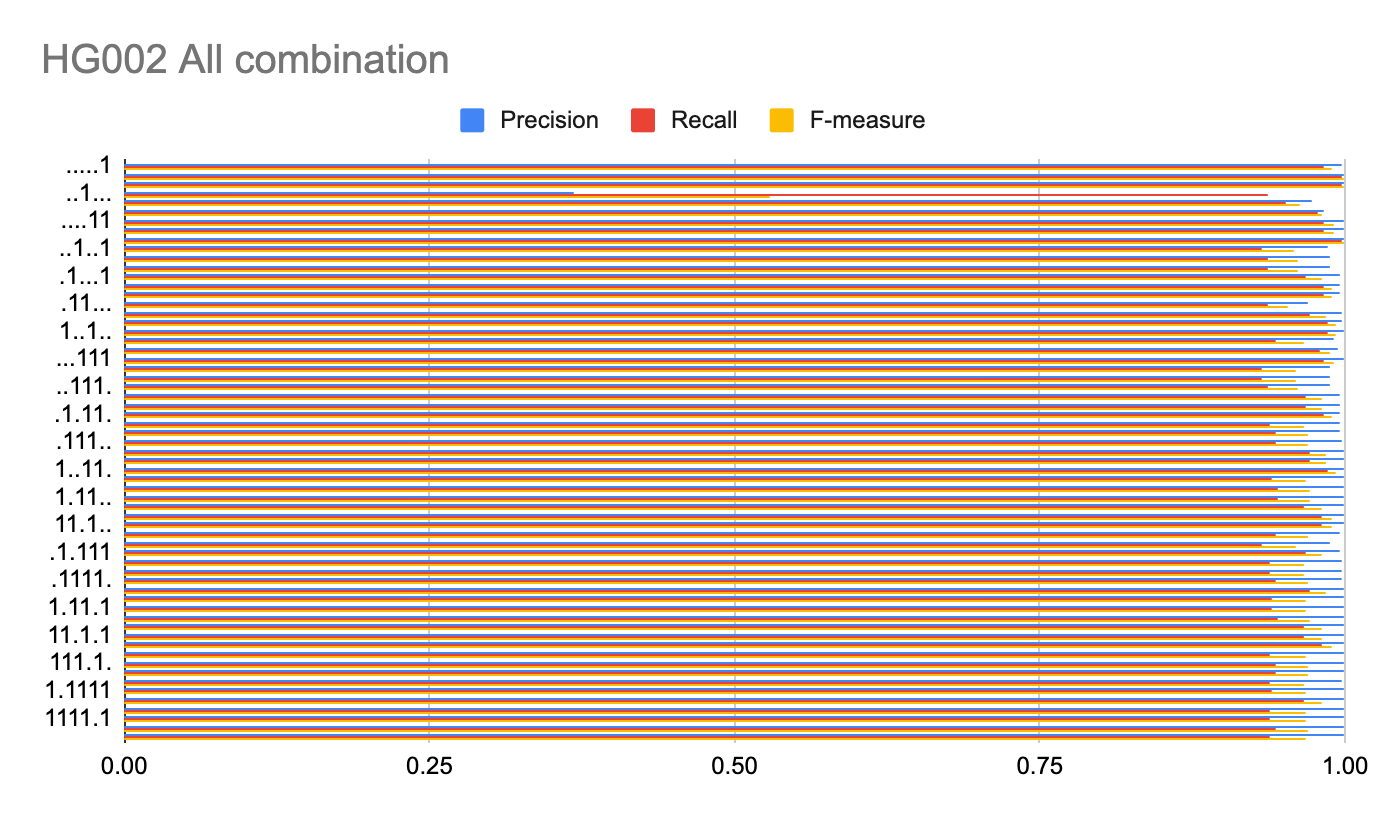


Figure 4: In this figure, the benchmark sample HG002 SNVs are plotted on the x-axis, with measures of precision (blue), recall (yellow), and F-score (red) ranging from 0 to 1. On the y-axis is a permutation of each combination of SNV callers (Longshot, Clair, and DeepVariant) for both PacBio HiFi and ONT data. A value of 1 indicates the presence of a given combination, while a value of "." indicates its absence. The combinations are sorted as follows: ONT-DeepVariant, ONT-Clair, ONT-Longshot, HiFi-DeepVariant, HiFi-Clair, and HiFi-Longshot.


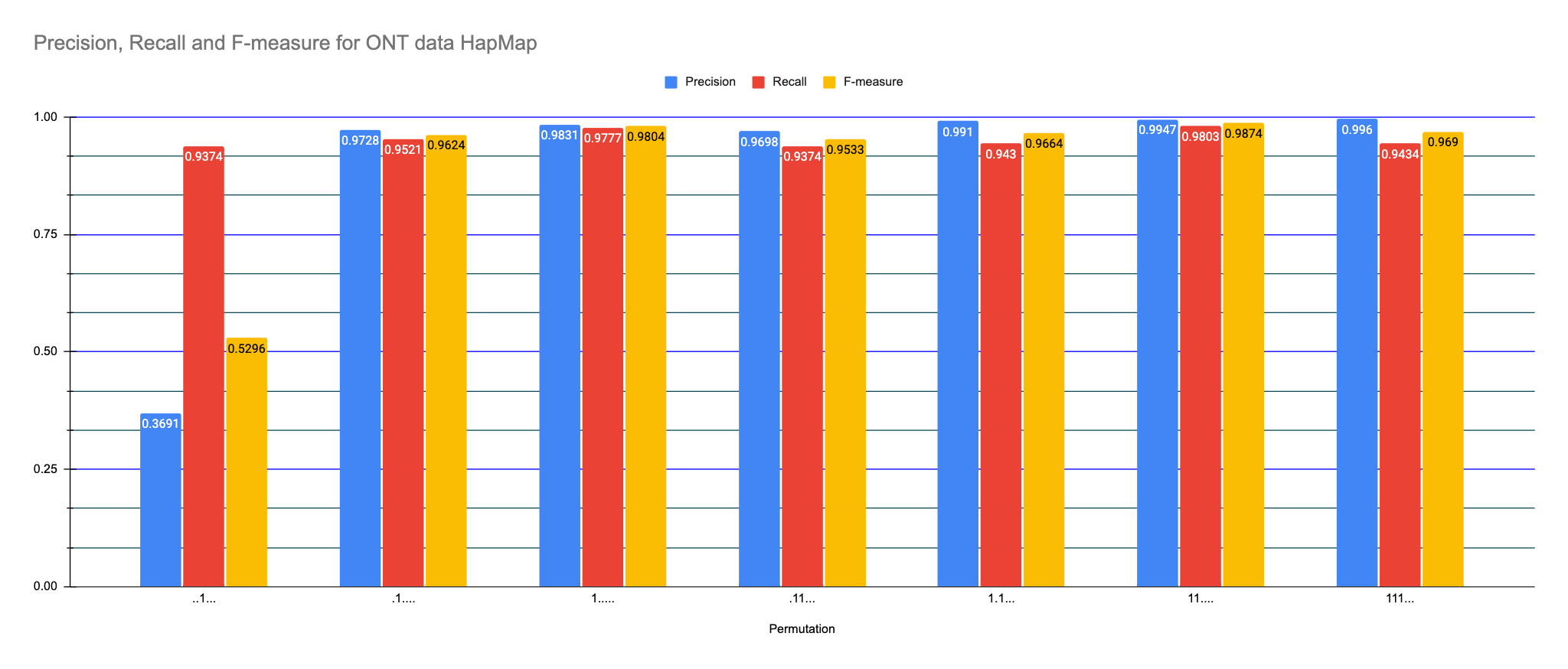


Figure 5: In this figure, the benchmark sample HG002 ONT SNVs are plotted on the x-axis as a permutation of each combination of SNV callers (Longshot, Clair, and DeepVariant) for both PacBio HiFi and ONT data. A value of 1 indicates the presence of a given combination, while a value of "." indicates its absence. The combinations are sorted as follows: ONT-DeepVariant, ONT-Clair, ONT-Longshot, HiFi-DeepVariant, HiFi-Clair, and HiFi-Longshot. On the y-axis, measures of precision (blue), recall (yellow), and F-score (red) are plotted, ranging from 0 to 1.


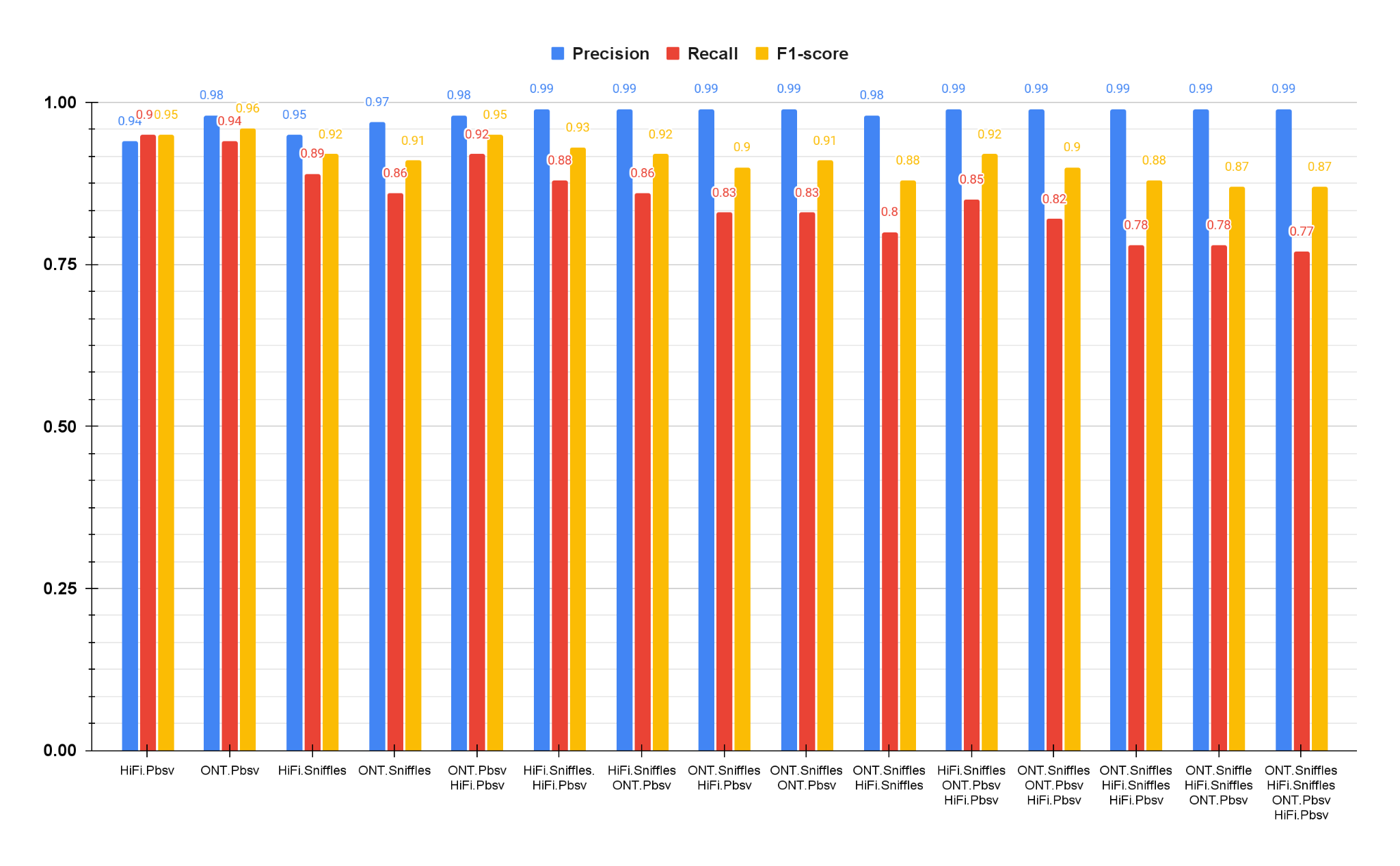


Figure 6: The precision, recall, and F-score for structural variants (SVs) in medical regions are compared for different combinations of callers (Sniffles and Pbsv) and technologies for the HG002 sample.


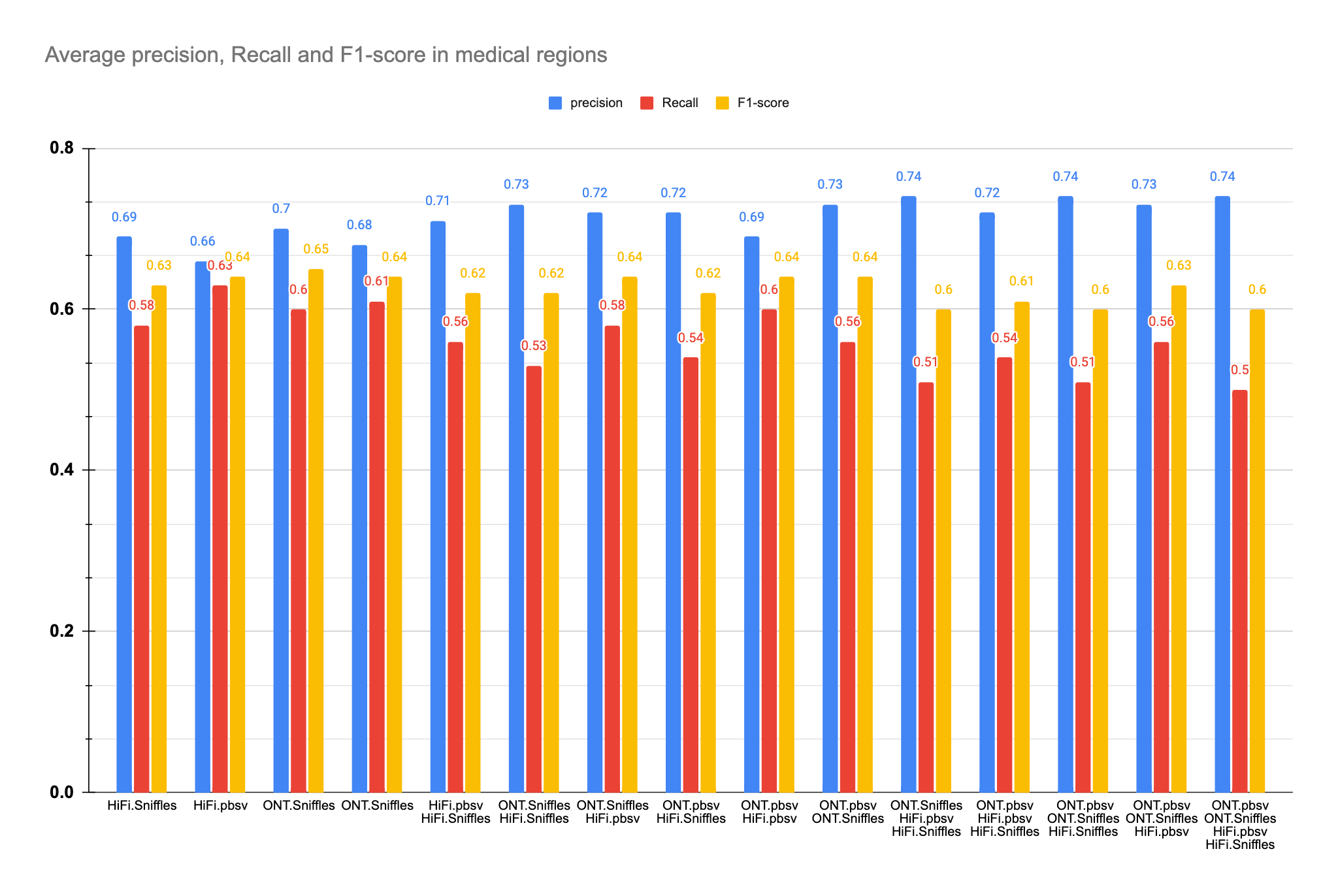


Figure 7: The genome-wide average precision, recall, and F-score are compared between HapMap samples (HG00514, HG00733, and NA19240) for different callers and technologies.


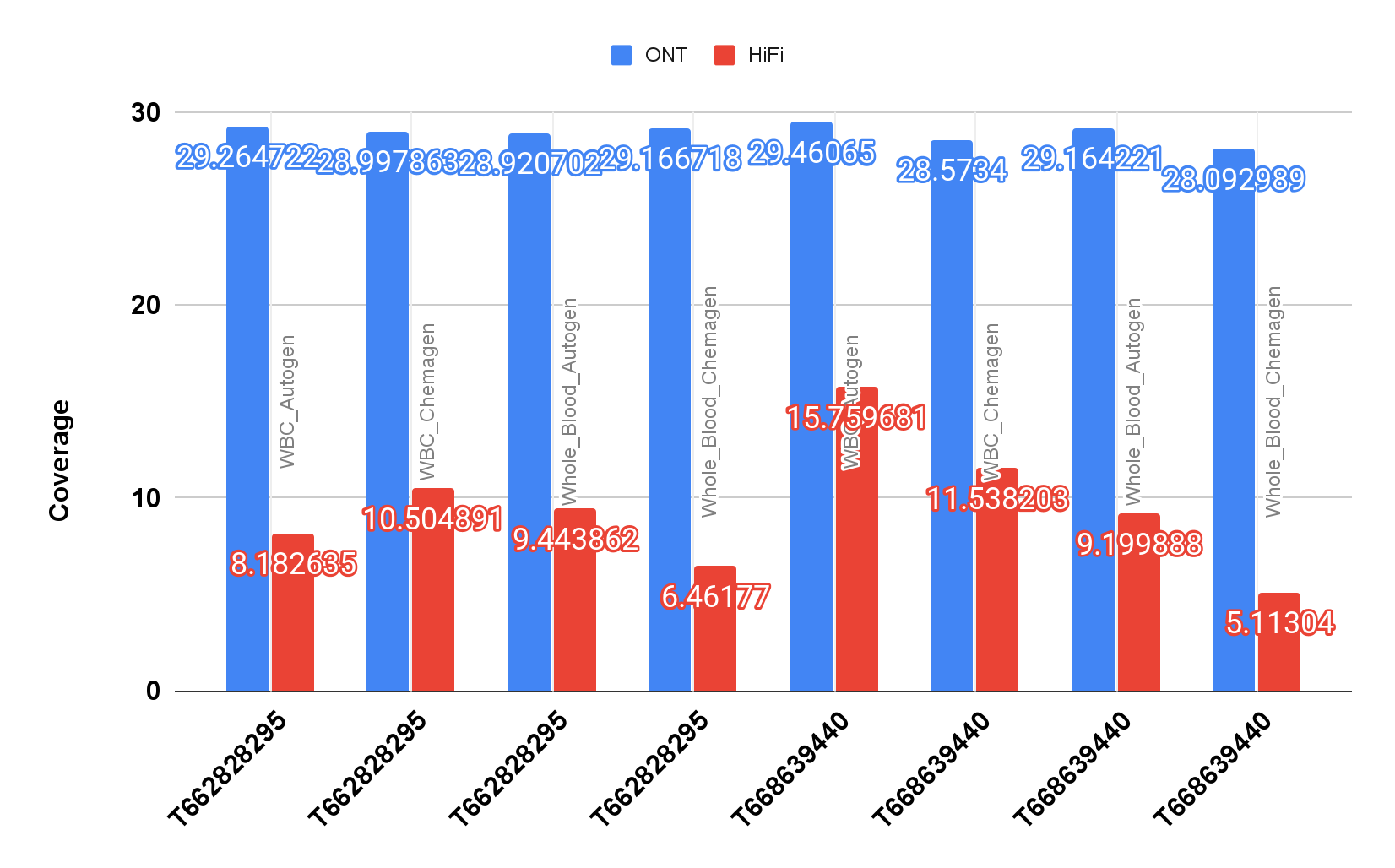


Figure 8: Coverage comparison between HiFi and ONT in two samples using different tissue and extraction methods.


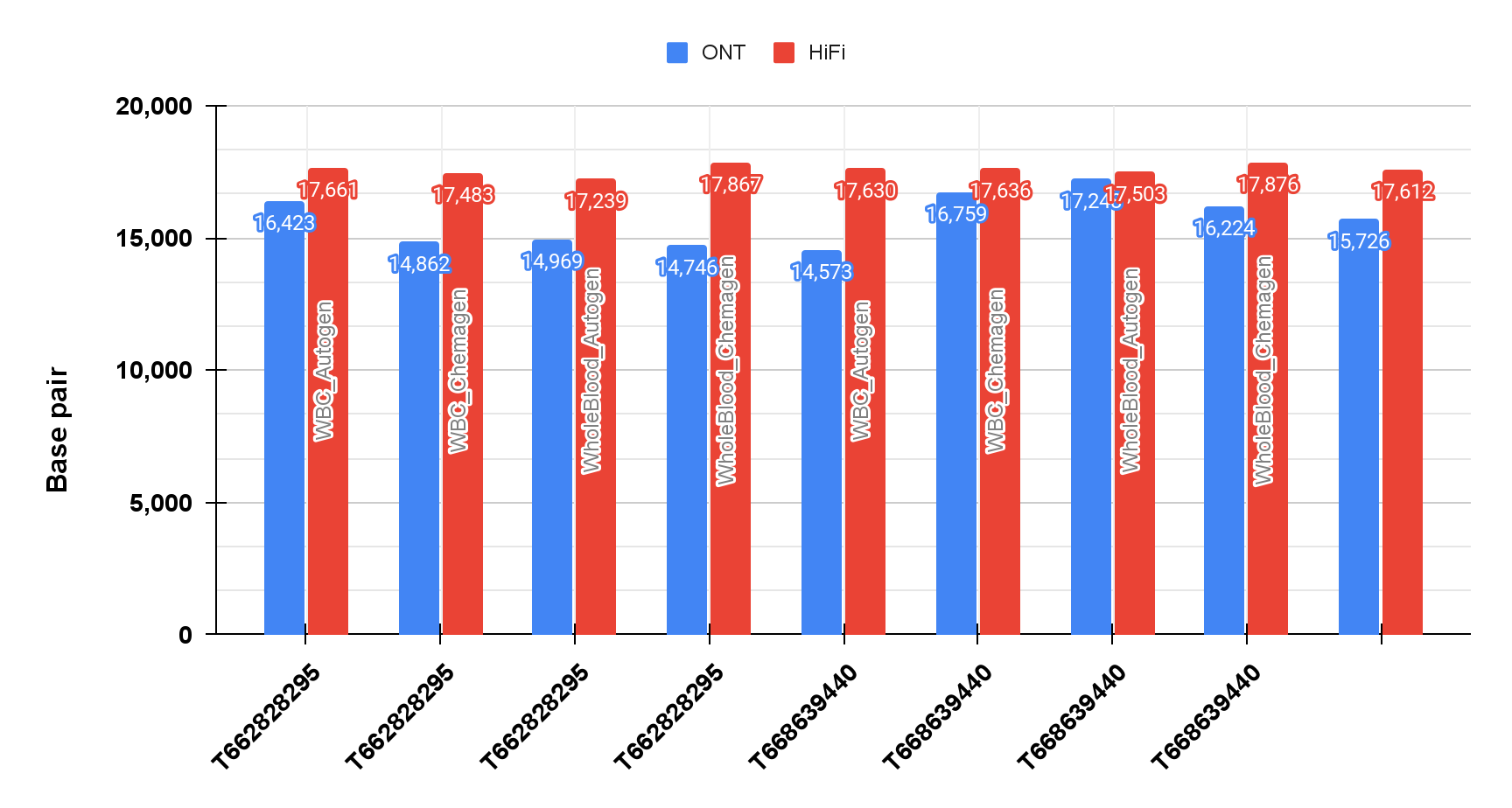


Figure 9: Aligning N50 between HiFi and ONT in two samples using different tissue and extraction methods.


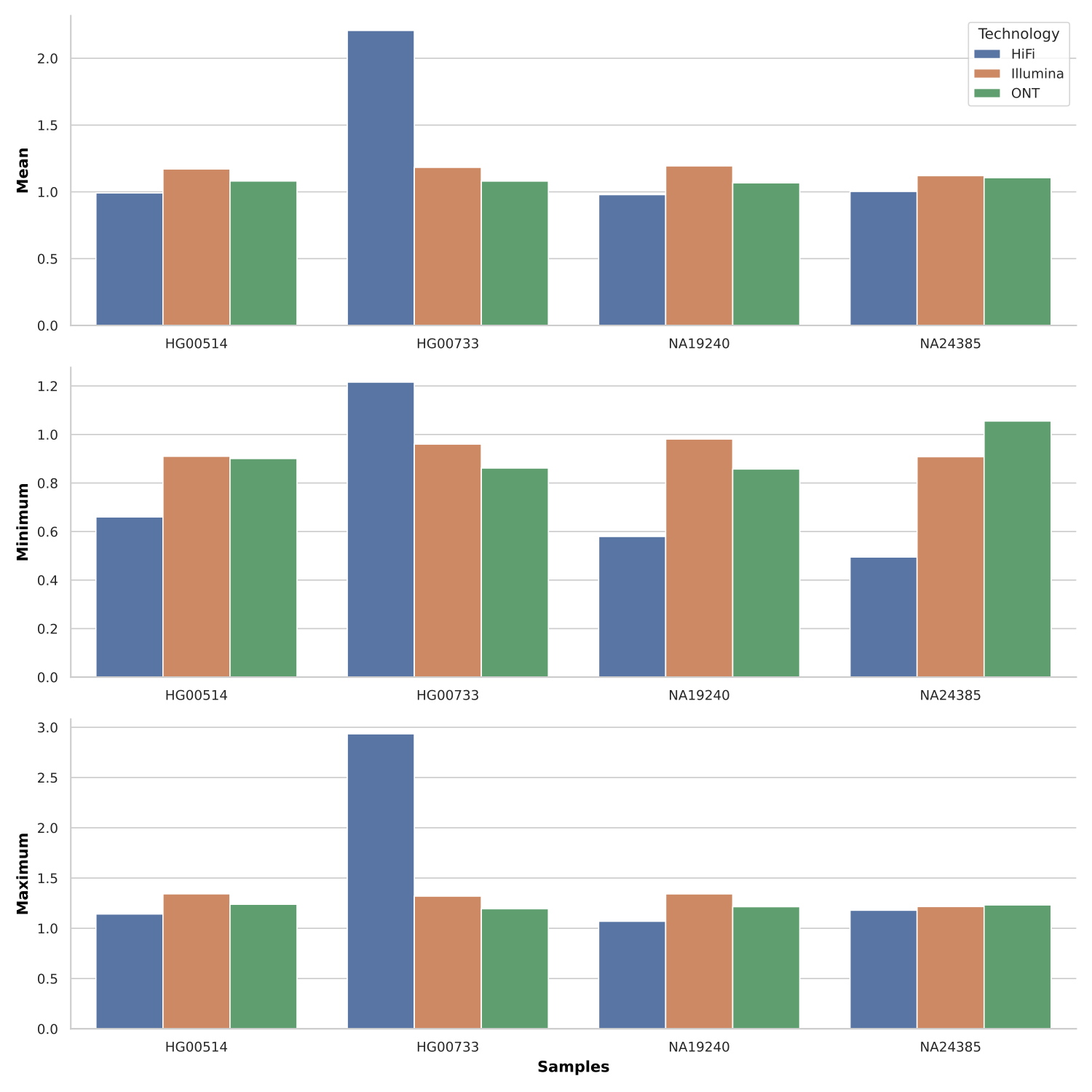


Figure 10: Mean, maximum, and minimum normalized coverage for the ACMG genes group within the 4,641 genes (68 genes).


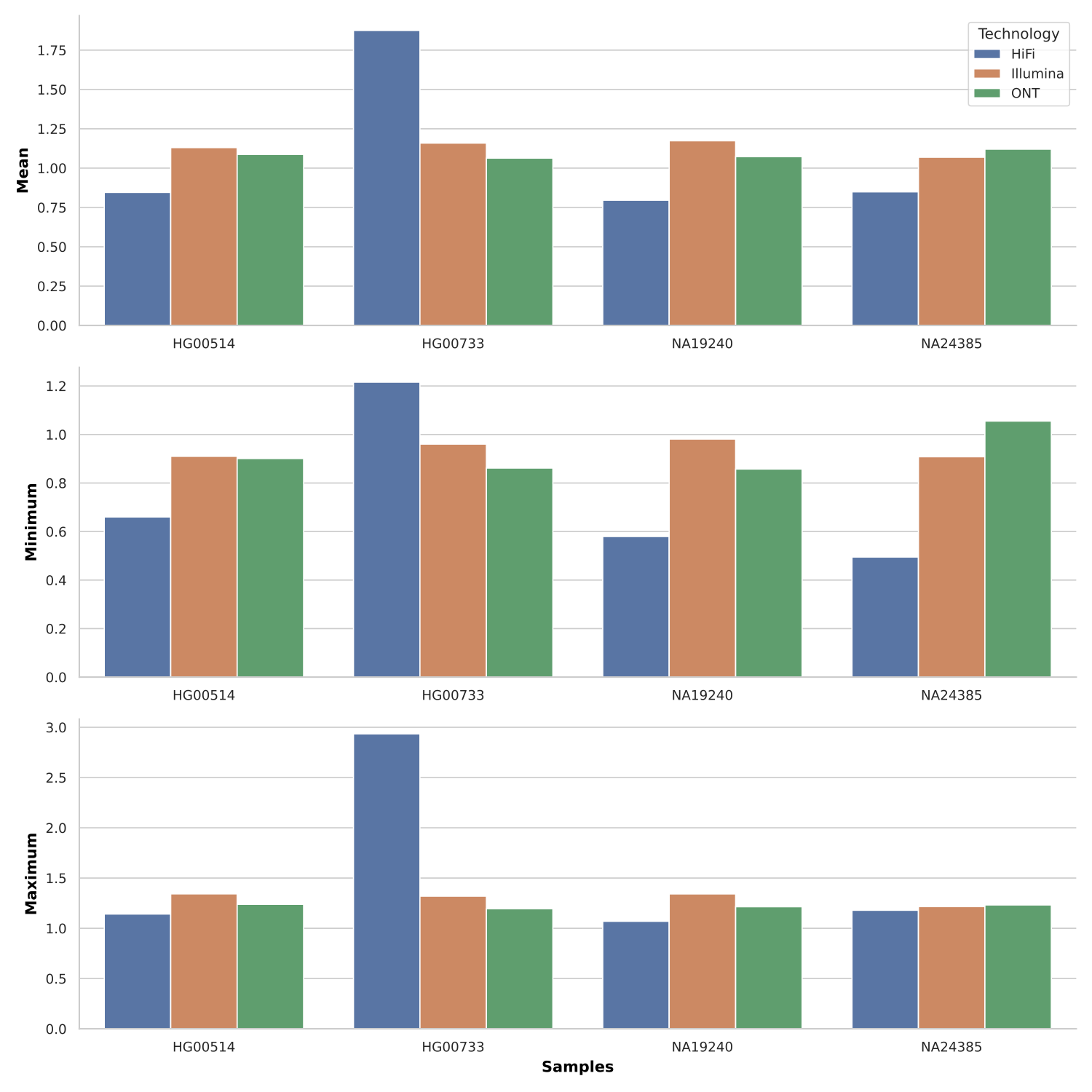


Figure 11: Mean, maximum, and minimum normalized gene coverage for the ACMG gene group within 386 genes (5 genes).


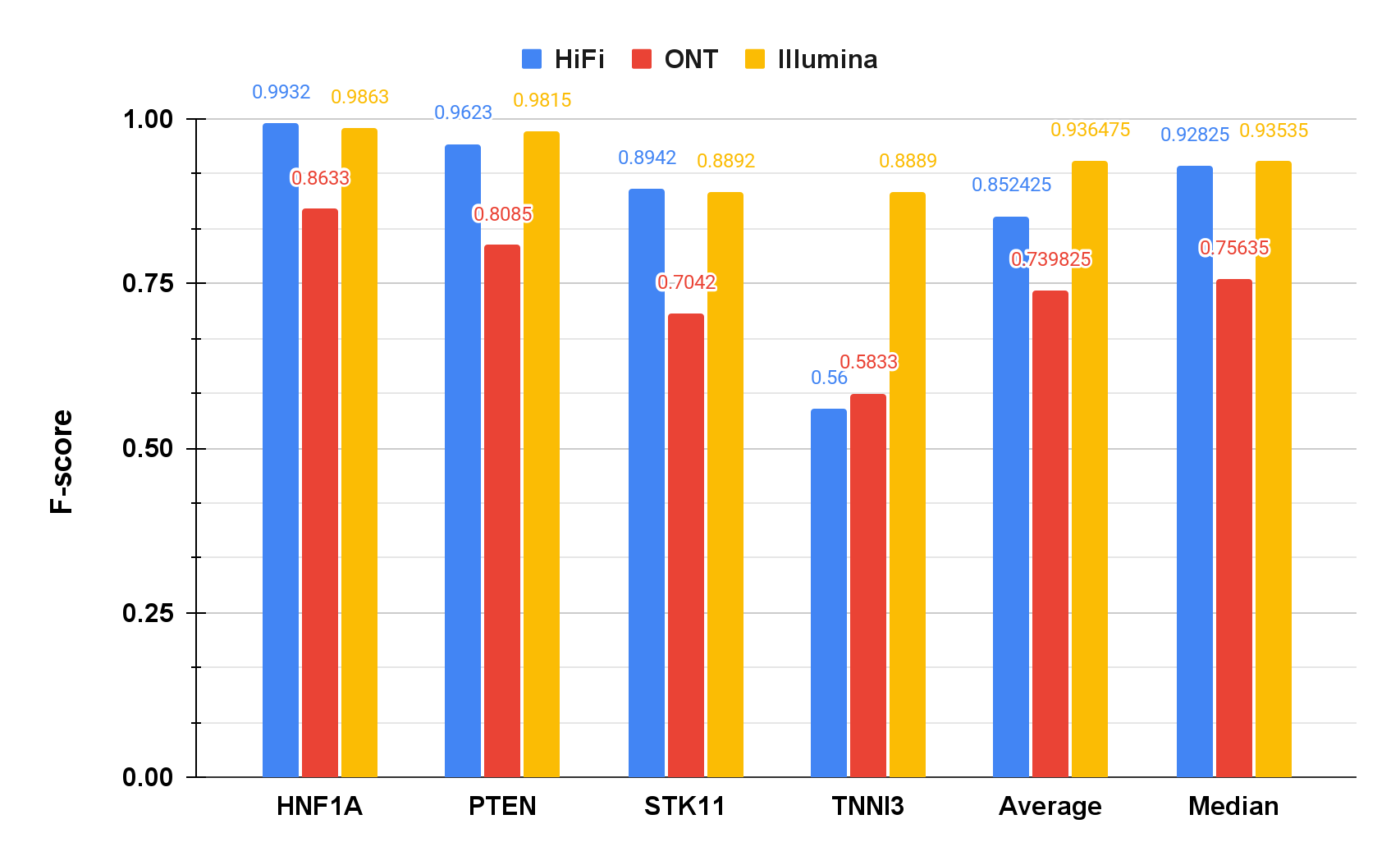


Figure 12: Comparison of f-score for each technology in GIAB Challenging medically relevant genes (CMRG).


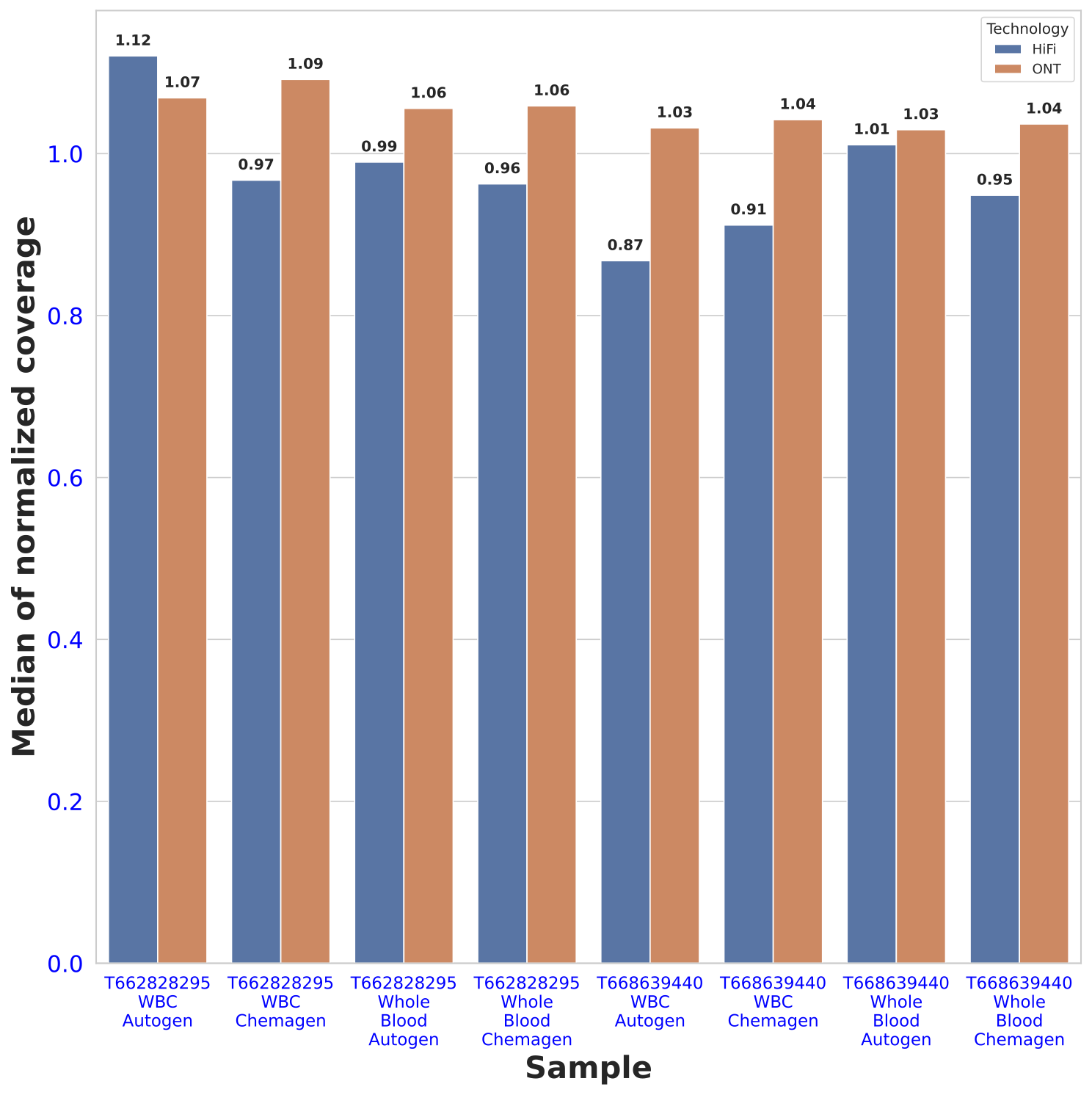


Figure 13: Median normalized coverage comparison between HiFi (blue) and ONT (orange) for eight datasets on the x-axis, with the median coverage on the y-axis.


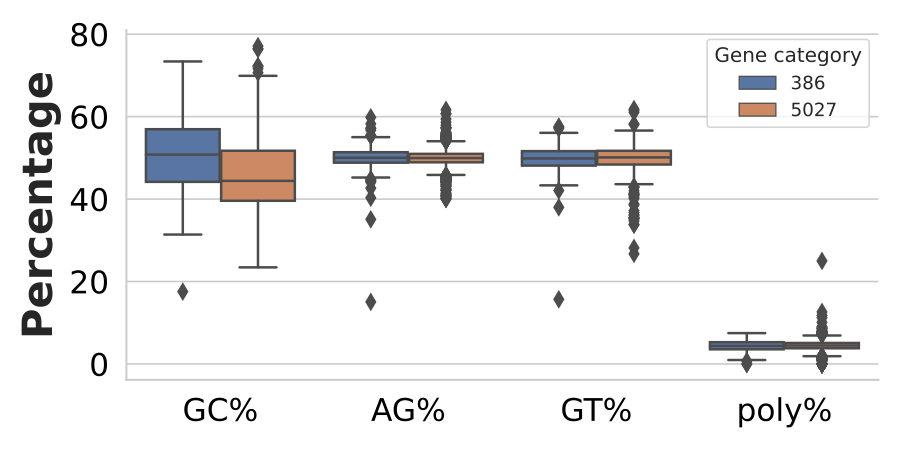


Figure 14: Percentage of guanine (G), cytosine (C), adenine (A), and thymine (T) bases, as well as homopolymer bases, in the two gene sets.


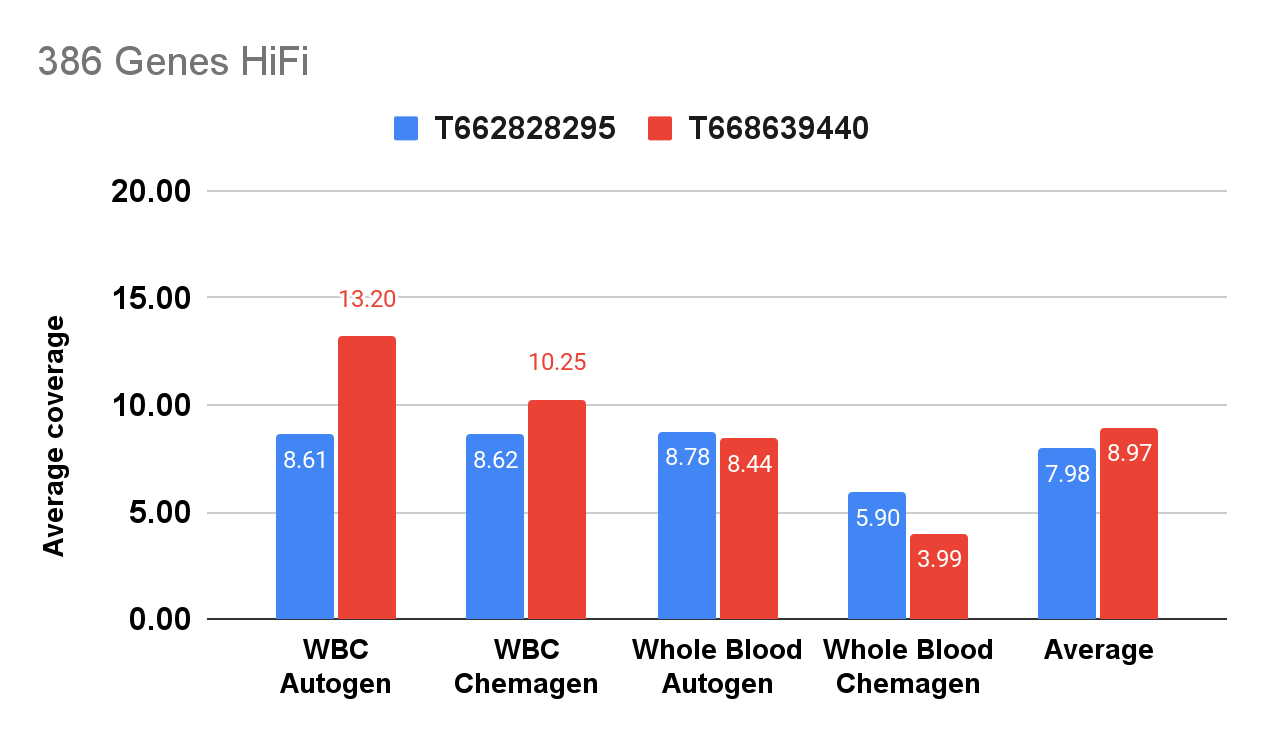


Figure 15: The average variant coverage per gene is shown for samples T662828295 and T668639440 using PacBio HiFi, specifically for the challenging group of 386 genes.


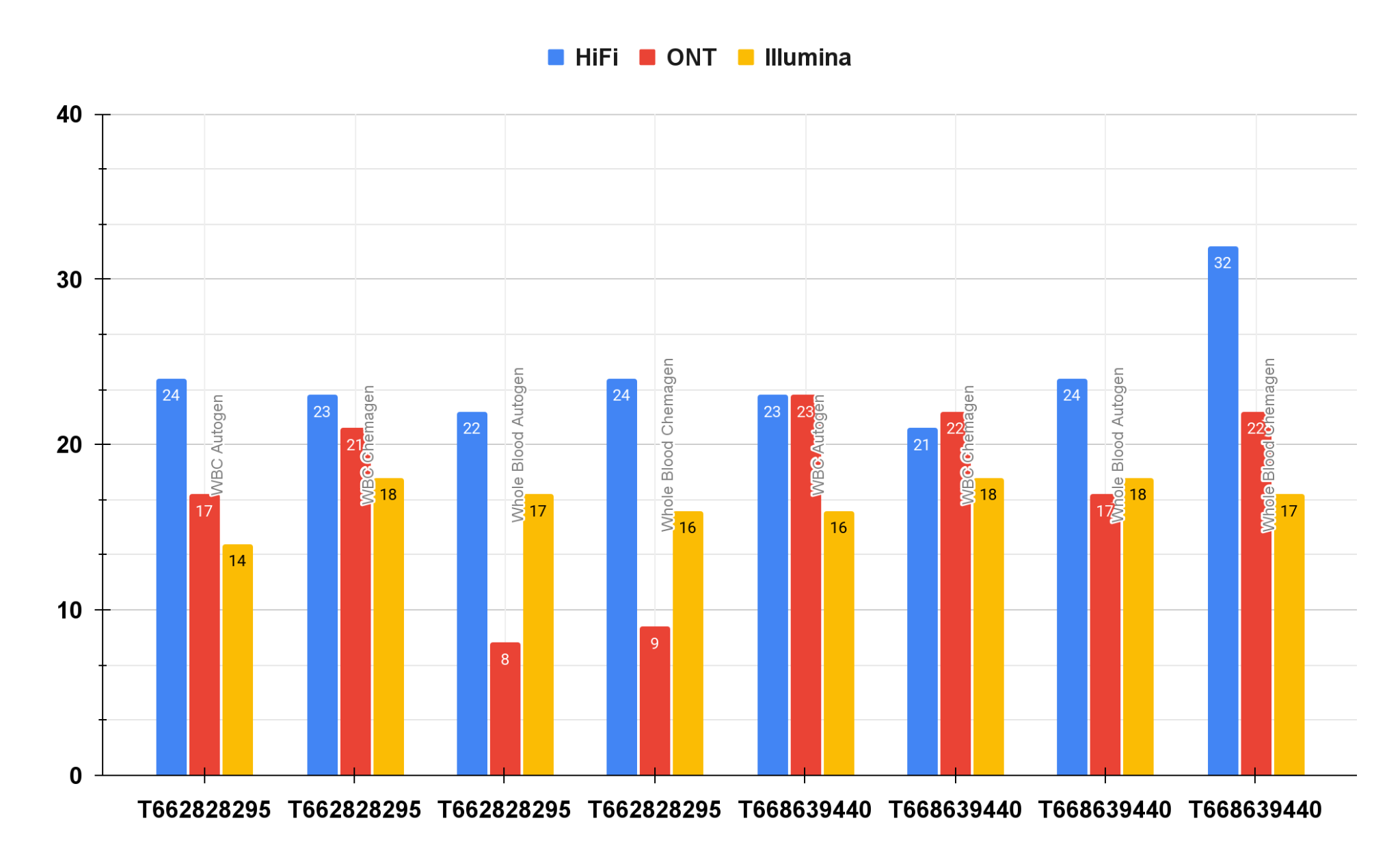


Figure 16:The number of uncovered pathogenic variants for PacBio HiFi, Illumina, and ONT technologies is shown for different tissue sources and extraction methods across eight datasets.


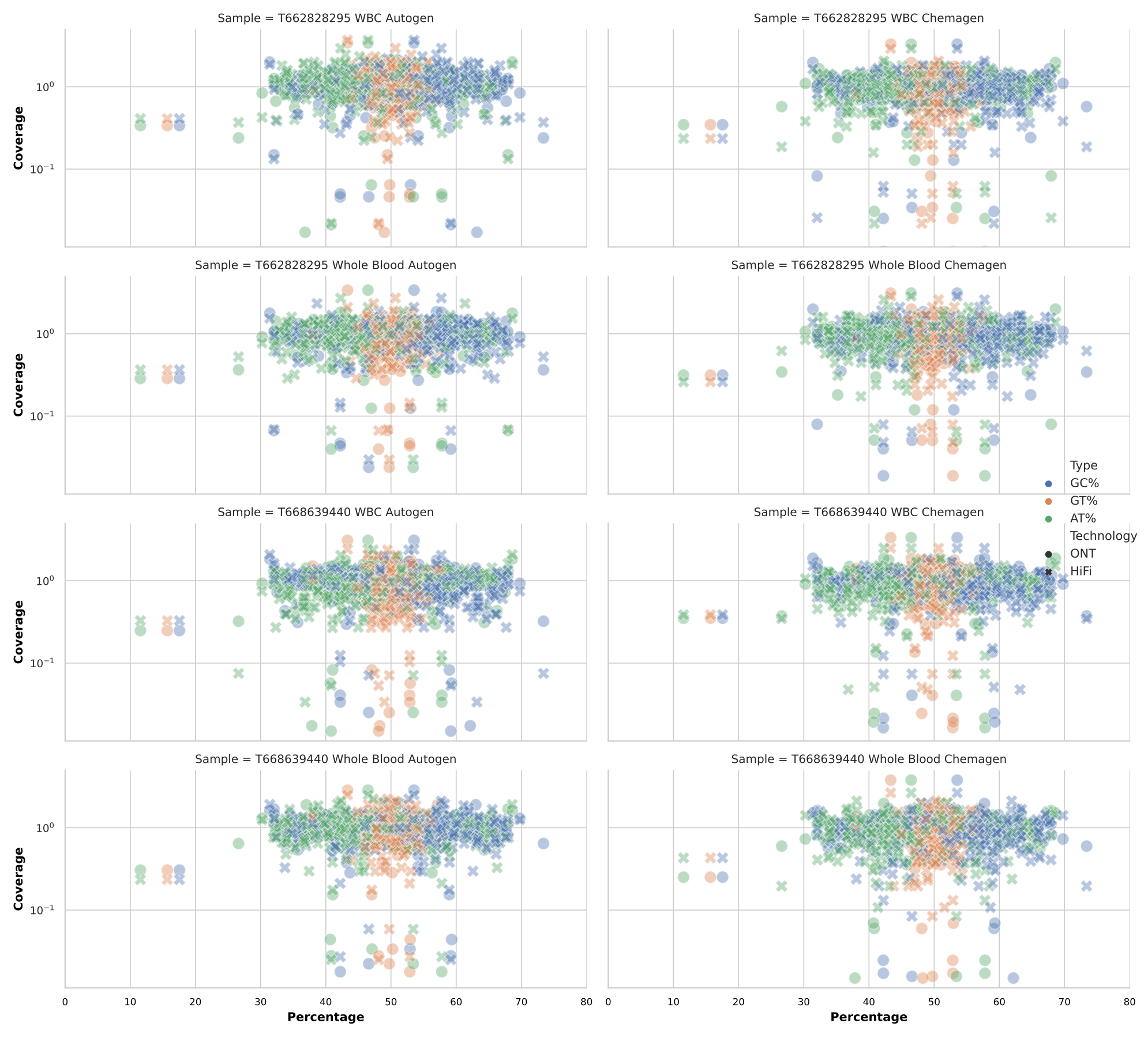


Figure 17: On the x-axis is the percentage of GC, GT, and AT bases (represented by blue, orange, and green, respectively) plotted against the log-normalized gene coverage for ONT (represented by circles) and HiFi (represented by x's). Each panel represents a different tissue source and extraction method.


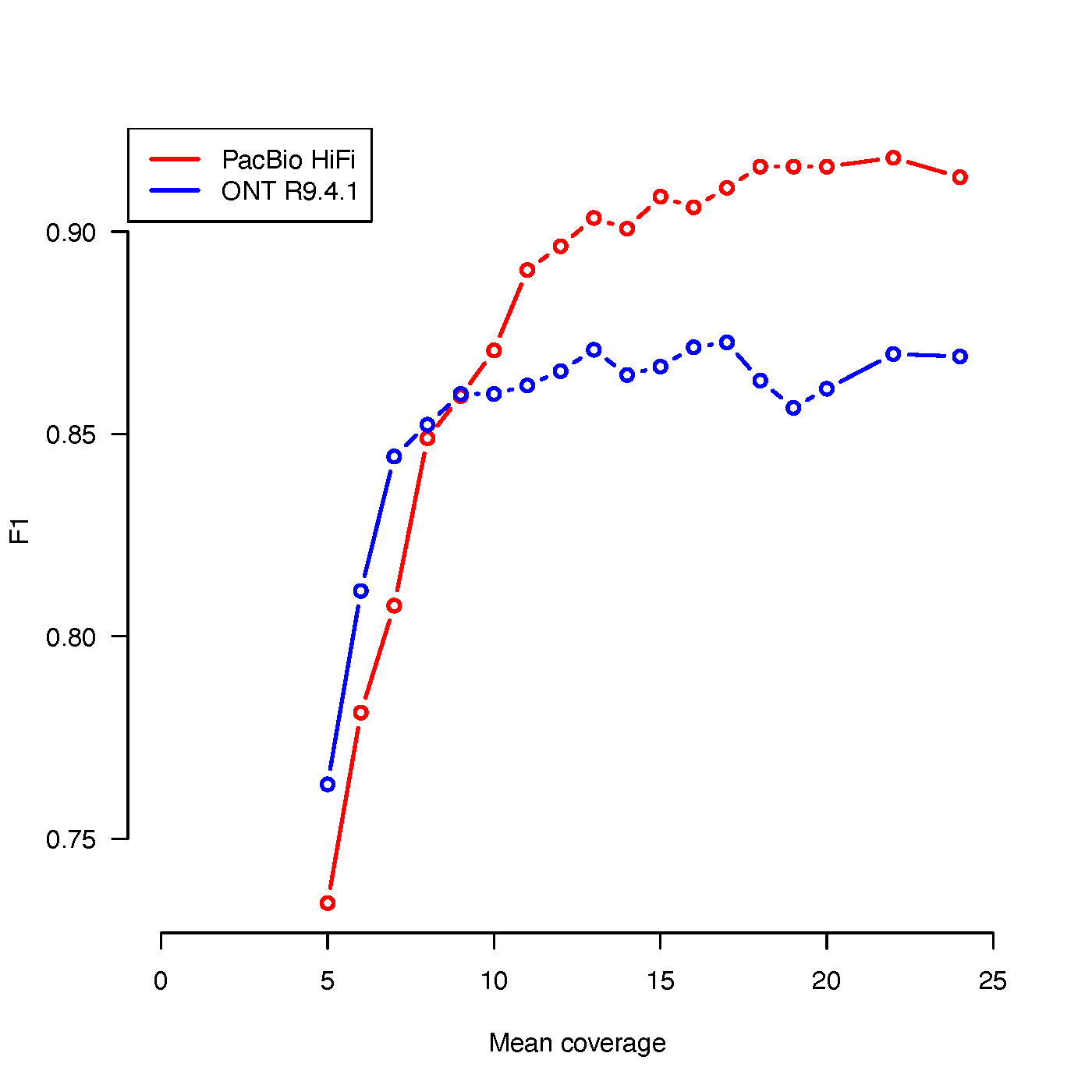


Figure 18: A comparison of SV F1-score on the Y-axis for ONT and HiFi at different average genome coverage levels on the X-axis.


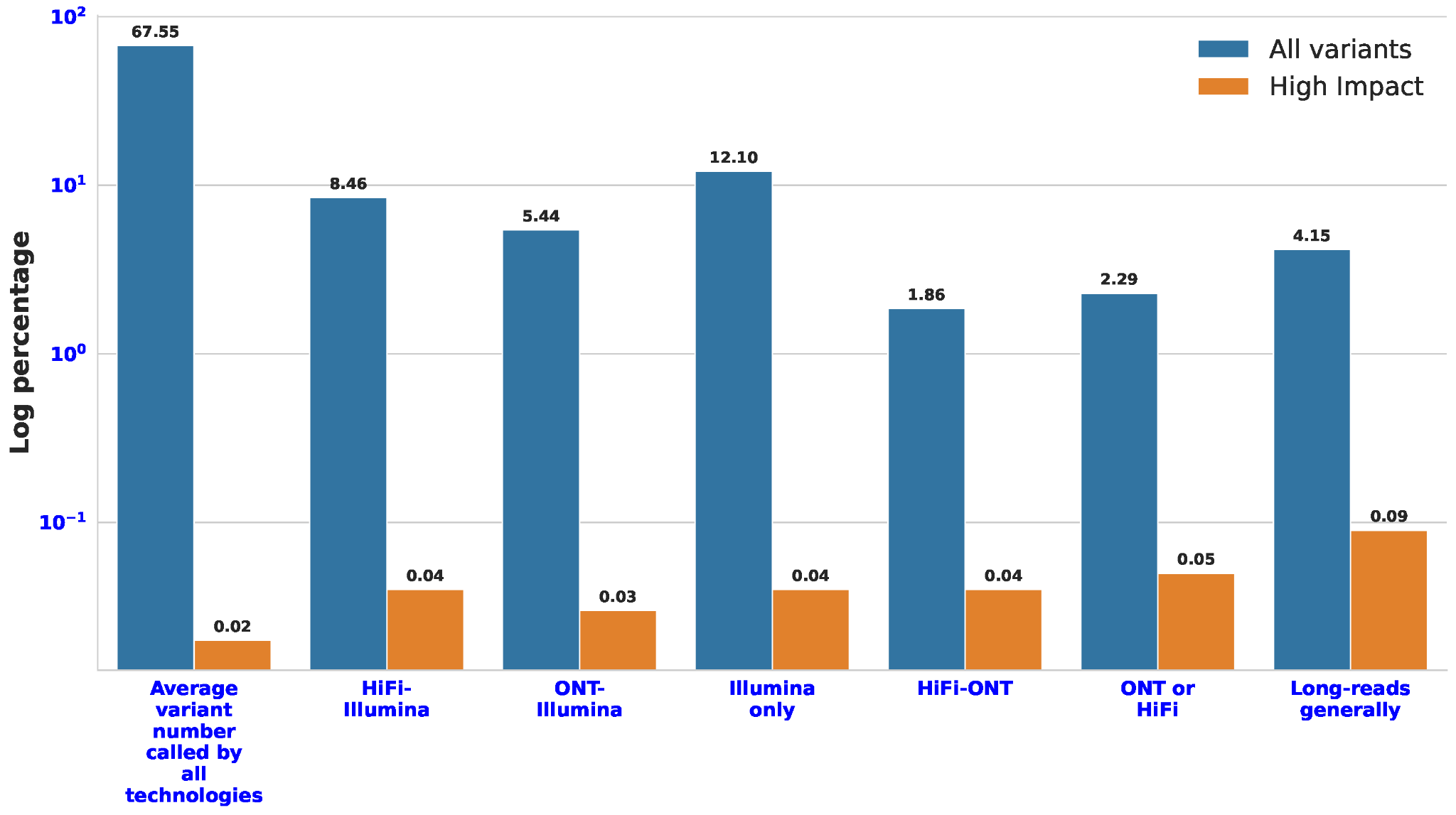


Figure 18: A comparison of the percentage of high-impact variants (SNVs and indels) on the Y-axis, categorized by each technology on the X-axis. This includes variants detected by multiple technologies, as well as variants identified by a single technology.


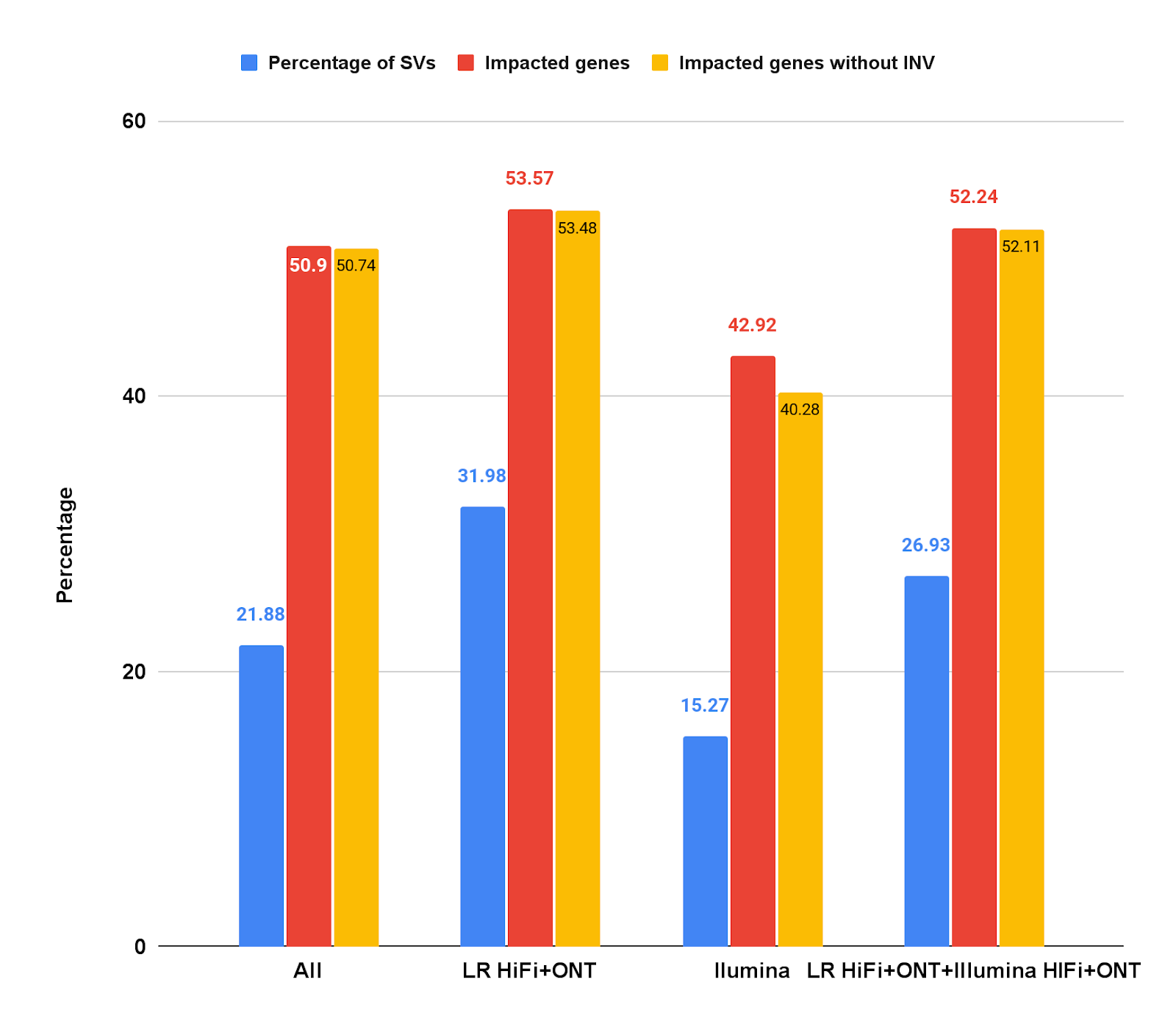


Figure 19: A comparison of the percentage of impacted genes on the Y-axis, categorized by each technology on the X-axis. This includes SVs detected by multiple technologies, as well as SVs identified by a single technology.
