# Supplementary material for: Utility of long-read sequencing for All of Us
Source: Nat Commun. 2024 Jan 29;15:837. doi: 10.1038/s41467-024-44804-3 (PMC10822842; doi:10.1038/s41467-024-44804-3)
Supplement: Supplementary file 3 — Description of Additional Supplementary Files [file 41467_2024_44804_MOESM3_ESM.pdf]

Description of Additional Supplementary Files

File Name: Supplementary Data 1
Description: The table presents the coverage and N50 values for PacBio HiFi, Oxford Nanopore Technologies, and Illumina sequencing technologies. The analysis includes multiple samples, namely HG00514, HG00733, NA19240, NA24385, T662828295, and T668639440.

File Name: Supplementary Data 2
Description: Comparison of single nucleotide variants (SNVs), insertions, and deletions identified in samples T662828295 and T668639440 for different tissues: white blood cells (WBC) and whole blood cells. The extractions methods used were Autogen and Chemagen. The table illustrates the unique variants and shared variants between these samples, along with their respective percentages.

File Name: Supplementary Data 3
Description: Comparison of single nucleotide variants (SNVs), insertions, and deletions identified in the exome and intron regions of easy access genes in samples T662828295 and T668639440, separately, for different tissues: white blood cells (WBC) and whole blood cells. The extraction methods used were Autogen and Chemagen. The table illustrates the unique variants and shared variants between these samples, along with their respective percentages.

File Name: Supplementary Data 4
Description: Average coverage per gene and number of uncovered variants per sample for both challenging and easy access medically relevant genes using PacBio HiFi, Oxford Nanopore Technologies, and Illumina technologies. The samples analyzed are T662828295 and T668639440.

File Name: Supplementary Data 5
Description: Average normalized coverage for the American College of Medical Genetics and Genomics (ACMG) gene set and the F-score ratio of the genes that intersect with the challenging medically relevant genes we analyzed.

File Name: Supplementary Data 6
Description: Genes with average coverage < 1 in Illumina for samples NA24385, HG00514, HG00733, and NA19240, and the number of these genes that are uncovered across samples for both challenging medically relevant genes and easy access genes. Additionally, the genes with average coverage less than 1 for challenging medically relevant genes between Samples T668639440 and T662828295 across the three technologies.

File Name: Supplementary Data 7
Description: In this sheet, we are comparing the performance of Oxford Nanopore Technologies (ONT), HiFi, and Illumina by benchmarking 273 genes from GIAB. This includes genes where at least one technology did not call any variants, some of which may be due to the benchmark set itself not containing any variants. Additionally, we are comparing the highest 10 genes' F-score and the lowest 10 F-score per technology, along with how the other technologies performed on those genes.

File Name: Supplementary Data 8
Description: Comparison of single nucleotide variants (SNVs), insertions, and deletions identified in the exome and intron regions of easy access genes in samples T662828295 and T668639440, separately, for different tissues: white blood cells (WBC) and whole blood cells. The extraction methods used were Autogen and Chemagen. The table illustrates the unique variants and shared variants between these samples, along with their respective percentages.
