# Supplementary material for: Utility of long-read sequencing for All of Us
Source: Nat Commun. 2024 Jan 29;15:837. doi: 10.1038/s41467-024-44804-3 (PMC10822842; doi:10.1038/s41467-024-44804-3)
Supplement: Supplementary file 5 — Reporting Summary [file 41467_2024_44804_MOESM5_ESM.pdf]

nature portfolio

Corresponding author(s): Fritz J Sedlazeck

Last updated by author(s): 12/12/2023

Reporting Summary

Nature Portfolio wishes to improve the reproducibility of the work that we publish. This form provides structure for consistency and transparency in reporting. For further information on Nature Portfolio policies, see our Editorial Policies and the Editorial Policy Checklist.

Statistics

For all statistical analyses, confirm that the following items are present in the figure legend, table legend, main text, or Methods section.

n/a	Confirmed	
☐	☒	The exact sample size (*n*) for each experimental group/condition, given as a discrete number and unit of measurement
☒	☐	A statement on whether measurements were taken from distinct samples or whether the same sample was measured repeatedly
☒	☐	The statistical test(s) used AND whether they are one- or two-sided<br>*Only common tests should be described solely by name; describe more complex techniques in the Methods section.*
☒	☐	A description of all covariates tested
☒	☐	A description of any assumptions or corrections, such as tests of normality and adjustment for multiple comparisons
☐	☒	A full description of the statistical parameters including central tendency (e.g. means) or other basic estimates (e.g. regression coefficient) AND variation (e.g. standard deviation) or associated estimates of uncertainty (e.g. confidence intervals)
☒	☐	For null hypothesis testing, the test statistic (e.g. *F*, *t*, *r*) with confidence intervals, effect sizes, degrees of freedom and *P* value noted<br>*Give P values as exact values whenever suitable.*
☒	☐	For Bayesian analysis, information on the choice of priors and Markov chain Monte Carlo settings
☒	☐	For hierarchical and complex designs, identification of the appropriate level for tests and full reporting of outcomes
☒	☐	Estimates of effect sizes (e.g. Cohen's *d*, Pearson's *r*), indicating how they were calculated

Our web collection on statistics for biologists contains articles on many of the points above.

Software and code

Policy information about availability of computer code

Data collection	We downloaded the mappability track using wget ftp://ftp-trace.ncbi.nlm.nih.gov/ReferenceSamples/giab/release/genome-stratifications/v2.0/GRCh38/mappability/GRCh38_lowmappabilityall.bed.gz<br>We collected pathogenic variant from ClinVar using wget https://ftp.ncbi.nlm.nih.gov/pub/clinvar/vcf_GRCh38/clinvar_20220320.vcf.gz<br>Samples are accessible thought AllOfUs: https://workbench.researchallofus.org/
Data analysis	Plots were generated with Python (version 3.7.10) and Seaborn (version 0.11.2).<br>PacBio data were aligned with pbmm2 (1.4.0) with the parameters (--preset ccs --strip --sort --unmapped); MD tag are then added by the samtools calmd command (samtools 1.10).<br>ONT data were aligned with minimap2 (2.17-r941).<br>Aligned bams of each flow-/SMRT-cell are then merged by sample with samtools merge (samtools 1.10).<br>We calculated coverage at the sample level using mosdepth (0.3.1).Per gene average coverage also collected by mosdepth.<br>Additionally, we calculated per-base coverage using samtools depth (1.15.1) with the parameters (-a).<br>For small variant calling we used Clair343 (v0.1-r6) for each sample, calls are made per chromosome with default parameters, and then merged, and followed with bcftools sort (bcftools 1.13).<br>We additionally applied PEPPER-Margin-DeepVariant (version r0.4.1) for CCS data.<br>For phasing we used MarginPhase (from docker image kishwars/pepper_deepvariant:r0.4.1).<br>We used Sniffles (1.0.12) and PBSV (2.6.0) for SV calls.<br>Illumina data analysis was done using the Dragen pipeline (v3.4.12) and called SVs using Manta (v1.6.0) .<br>To calculate mappability we used bedtools (version 2.30.0) intersect -wo where -a is the genes and -b is the mappability track, and sum all intersect lengths within the gene divided by gene length to calculator the gene mappability intersect percentage.<br>To select SNVs we used bcftools view -H -v snps, bcftools view -i 'strlen(REF)<strlen(ALT)' -H -v indels` for insertions, and `bcftools view -i

'strlen(REF)>strlen(ALT)' -H -v indels` for deletions.
To benchmark SNVs and indels we used RTG tools version 3.12.1.
and to filter SVs we used bcftools view -Oz -i '(SVLEN>=50 | SVLEN<=-50 | SVLEN=0 | SVLEN=1 | SVLEN=".") & (FILTER="PASS")'
We merged the identified SVs from technologies (HiFi and ONT) and tools (pbsv and Sniffels) using SURVIVOR (Version: 1.0.7) `SURVIVOR merge` with the following parameters `1000 1 1 0 0 50`.
We selected only pathogenic variant that have a provided criterion, multiple submitters, and no conflict using bcftools `bcftools view -i 'CLNSIG="Pathogenic" & CLNREVSTAT="criteria_provided\,_multiple_submitters\,_no_conflicts" & CHROM!="X" & CHROM !="MT"'`

For manuscripts utilizing custom algorithms or software that are central to the research but not yet described in published literature, software must be made available to editors and reviewers. We strongly encourage code deposition in a community repository (e.g. GitHub). See the Nature Portfolio guidelines for submitting code & software for further information.

Data

Policy information about availability of data

All manuscripts must include a data availability statement. This statement should provide the following information, where applicable:

- Accession codes, unique identifiers, or web links for publicly available datasets
- A description of any restrictions on data availability
- For clinical datasets or third party data, please ensure that the statement adheres to our policy

All data are available through the All of Us workbench https://www.researchallofus.org/data-tools/workbench/

Human research participants

Policy information about studies involving human research participants and Sex and Gender in Research.

Reporting on sex and gender

Use the terms sex (biological attribute) and gender (shaped by social and cultural circumstances) carefully in order to avoid confusing both terms. Indicate if findings apply to only one sex or gender; describe whether sex and gender were considered in study design whether sex and/or gender was determined based on self-reporting or assigned and methods used. Provide in the source data disaggregated sex and gender data where this information has been collected, and consent has been obtained for sharing of individual-level data; provide overall numbers in this Reporting Summary. Please state if this information has not been collected. Report sex- and gender-based analyses where performed, justify reasons for lack of sex- and gender-based analysis.

Population characteristics

Describe the covariate-relevant population characteristics of the human research participants (e.g. age, genotypic information, past and current diagnosis and treatment categories). If you filled out the behavioural & social sciences study design questions and have nothing to add here, write "See above."

Recruitment

Describe how participants were recruited. Outline any potential self-selection bias or other biases that may be present and how these are likely to impact results.

Ethics oversight

Identify the organization(s) that approved the study protocol.

Note that full information on the approval of the study protocol must also be provided in the manuscript.

Field-specific reporting

Please select the one below that is the best fit for your research. If you are not sure, read the appropriate sections before making your selection.

☒ Life sciences ☐ Behavioural & social sciences ☐ Ecological, evolutionary & environmental sciences

For a reference copy of the document with all sections, see nature.com/documents/nr-reporting-summary-flat.pdf

Life sciences study design

All studies must disclose on these points even when the disclosure is negative.

Sample size	12 samples.
Data exclusions	Data were not excluded from analysis.
Replication	N/A
Randomization	N/A
Blinding	N/A

Reporting for specific materials, systems and methods

We require information from authors about some types of materials, experimental systems and methods used in many studies. Here, indicate whether each material, system or method listed is relevant to your study. If you are not sure if a list item applies to your research, read the appropriate section before selecting a response.

Materials & experimental systems

n/a	Involved in the study
☒	☐ Antibodies
☐	☒ Eukaryotic cell lines
☒	☐ Palaeontology and archaeology
☒	☐ Animals and other organisms
☒	☐ Clinical data
☒	☐ Dual use research of concern

Methods

n/a	Involved in the study
☒	☐ ChIP-seq
☒	☐ Flow cytometry
☒	☐ MRI-based neuroimaging

Eukaryotic cell lines

Policy information about cell lines and Sex and Gender in Research

Cell line source(s)	All samples (NA24385, HG00514, HG00733, and NA19240) were obtained from the Coriell Institute.
Authentication	*Describe the authentication procedures for each cell line used OR declare that none of the cell lines used were authenticated.*
Mycoplasma contamination	Cell lines were not tested for mycoplasma contamination but no indication of contamination was observed.
Commonly misidentified lines (See ICLAC register)	No commonly misidentified cell lines were used.

March 2021
